# Isolation of Antidepressants and Their Metabolites from Saliva Using Supported Liquid Extraction (SLE)

**DOI:** 10.3390/biomedicines11030708

**Published:** 2023-02-25

**Authors:** Ewelina Dziurkowska, Marek Wesolowski

**Affiliations:** Department of Analytical Chemistry, Medical University of Gdansk, Gen. J. Hallera 107, 80-416 Gdansk, Poland

**Keywords:** antidepressants, metabolites, saliva, supported liquid extraction, UHPLC, validation

## Abstract

The determination of antidepressant drugs and their metabolites in the body, mainly in the blood, allows for the monitoring of drug levels and their metabolism, helps identify drug interactions, and reduces the likelihood of increased side effects. Due to numerous inconveniences associated with collecting blood in patients, therapeutic drug monitoring (TDM) based on saliva sampling could significantly improve patient comfort. Therefore, the aim of this study was to develop a method for the simultaneous determination of selected antidepressants (amitriptyline, mianserin, duloxetine, mirtazapine, sertraline, citalopram, and venlafaxine) and their metabolites (N-desmethylmirtazapine, norsertraline, N-desmethylcitalopram, O-desmethylvenlafaxine) in human saliva using supported liquid extraction (SLE). Chlordiazepoxide was used as an internal standard. UHPLC coupled with DAD detection was used for the determinations. The proposed method was validated by determining its linearity for saliva concentrations in the range 10–1000 ng/mL. For all the analyzed compounds, a linear relationship between the analytical signal and analyte concentration was obtained (R^2^ > 0.99), with the intra- and inter-day precisions expressed as a coefficient of variation (% CV) below 15% in all tested cases. The study showed the usefulness of the proposed method for the isolation of antidepressant drugs and their metabolites in saliva patients’ samples.

## 1. Introduction

The determination of drug levels in the body allows for the observation of the drug’s absorption rate and indirectly helps determine the health status of the patient. In addition, monitoring the concentrations of the active ingredient and its metabolites provide an opportunity to determine how well drug metabolism is proceeding [1]. This can help prevent the appearance of toxic effects and drug interactions, and the severity of side effects. Therapeutic drug monitoring (TDM) plays an especially significant role in the case of psychotropic drugs, which not only have to be absorbed into the bloodstream in the right concentration but should also be able to penetrate the blood–brain barrier [1].

Since the 1960s, studies have been conducted to confirm the relationship between the concentration of psychotropic drugs in the body and the effect they produce [1]. While the total concentrations of absorbed free and protein-bound fractions of the drugs are usually determined in the blood, they can also be determined in saliva. The free fraction is responsible for the action of the substance and usually, in the case of psychotropic drugs, accounts for about 10% of the concentration determined in the blood [1]. Using saliva for drug level determination has considerable advantages: sample collection is noninvasive, can be performed solely by the patient without the need for qualified medical personnel, and can be carried out at home. Usually, sampling takes about 2 min. When using an appropriate technique, determination can be carried out in saliva even when the patient suffers from a dry mouth [2].

When trying to use saliva to analyze the levels of active substances for therapeutic drug monitoring (TDM), it is essential to have a standardized correlation between the concentration of a compound in the saliva and the blood [3]. Studies on the suitability of saliva as a diagnostic material for the determination of amitriptyline, a first-generation drug approved by the FDA in 1961 [4], were conducted in the 1970s and confirmed the existence of a correlation between the concentrations of the free fraction of amitriptyline in blood and saliva [5,6].

Similar studies have been conducted for duloxetine, mirtazapine, venlafaxine, and citalopram and their metabolites, finding statistically significant correlations between oral fluid and serum levels [7]. Another study on whole blood and saliva levels also confirmed correlations for venlafaxine and its metabolite [8], although not for the metabolites of citalopram and sertraline [9]. Their lack of reliable correlations between blood and saliva levels may have been due to the small group size and the fact that the assay was conducted in whole blood. Similarly, probably due to the small group size (eight patients), another study failed to fully confirm the presence of a correlation between venlafaxine concentrations in blood and saliva [10]. 

In Poland, there are no routine tests for the concentration of antidepressants in saliva. However, in a small number of court investigations, the concentration of psychoactive substances in saliva are measured, and on the basis of a correlation between levels in the blood and saliva, information is recorded on which concentration in the blood corresponds to the obtained concentration in saliva.

Antidepressants in saliva are most often determined chromatographically by gas chromatography (GC) [11,12] and liquid chromatography (LC) [7,8,9,10,13,14,15,16,17,18,19,20,21]. However, before chromatographic separation, it is necessary to isolate the analyte from the biological material. The most commonly used method is solid-phase extraction (SPE) [10,11,15,16,17,18,19], liquid–liquid extraction (LLE) [5,13,17], and more recently, microextraction by packed sorbent (MEPS) [9,21] and protein precipitation (PP) [7]. 

Classical liquid–liquid extraction (LLE) provides good sample purification and efficient isolation of lipophilic compounds; however, it is a time-consuming method. A modification of liquid–liquid extraction is supported liquid extraction (SLE), which offers the possibility of efficient sample purification and does not pose the risk of emulsifying during the process. The isolation of compounds is carried out in columns packed with a homogeneous form of diatomaceous earth. The procedure is relatively easy and can be automated. This type of extraction is primarily intended for the isolation of compounds with high lipophilicity from an aqueous matrix. During SLE, the aqueous sample is applied to the sorbent and the components to be analyzed are absorbed. The use of a suitable lipophilic solvent then causes desorption of the analytes. The collected solution is then evaporated for the dissolution of the dry residue in the mobile phase, and then is subjected to chromatographic analysis. 

SLE, like LLE, uses the same immiscible solvent systems for extraction. With SLE, when the aqueous sample is applied to the column, it disperses into small droplets which are immobilized on the cartridge, so there is no need to shake the sample, as is necessary when performing LLE. In addition, during SLE, the organic solvent (or a mixture of solvents) flowing through the sorbent leaches out the analyte, eliminating the possibility of emulsions forming, and greatly increasing extraction efficiency. 

To date, SLE has been used to determine substances with high lipophilicity, such as endogenous steroid hormones and endocannabinoids [22], eicosanoids [23], carotenoids (CAR), and fat-soluble vitamins [24]. The lipophilicity of antidepressants makes them good candidates for isolation from saliva by SLE. Moreover, saliva consists of about 99% water, which is another argument in favor of using SLE to isolate drugs from saliva [2]. 

**Figure 1:**
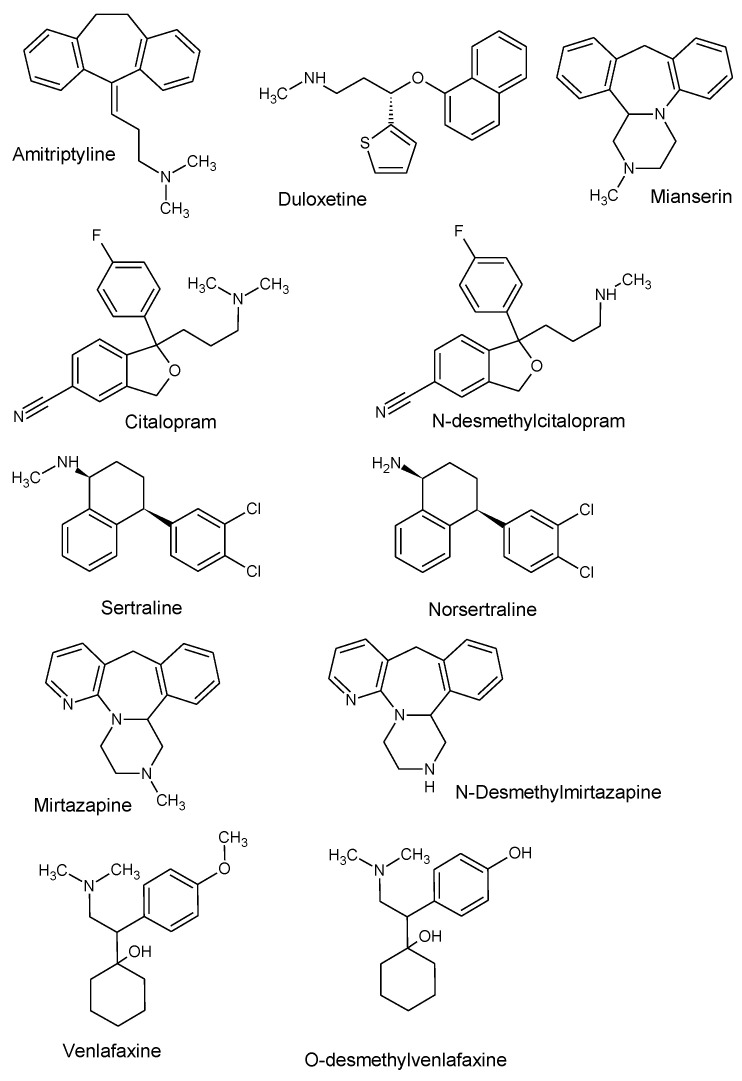
Chemical formulas of the analyzed substances and their metabolites.

SLE has been studied several times in isolating antidepressants, e.g., the determination of sertraline in tap water and wastewater [25], fluoxetine and its metabolite in plasma [26], and 13 antidepressants in blood samples [20]. SLE has not generally been used to isolate antidepressants from saliva, other than for the determination of psychoactive substances such as methylamphetamine, methylenedioxymethylamphetamine, and delta-9-tetrahydrocannabinol [27]. Therefore, the aim of this study was to develop and validate a rapid, sensitive, efficient, and selective method for isolating antidepressants (amitriptyline, mianserin, duloxetine, mirtazapine, sertraline, citalopram, and venlafaxine) along with their metabolites (N-desmethylmirtazapine, norsertraline, N-desmethylcitalopram, and O-desmethylvenlafaxine) from saliva samples using supported liquid extraction (SLE). We also tested whether the developed SLE method can be used to isolate the analytes under study from the saliva of patients treated with these compounds. The compounds selected for the study are among the most widely used antidepressant drugs in both mono- and polytherapy. The structural formulas of the parent compounds studied and their metabolites are listed in Figure 1.

## 2. Materials and Methods

### 2.1. Chemicals and Solvents

The following HPLC super-grade reagents were used in the study: acetonitrile (Merck, Darmstadt, Germany), dichloromethane (Supelco, Darmstadt, Germany), isopropanol, methanol, formic acid (FA), ammonia (POCh, Gliwice, Poland), and Ultra-Toc/UV purified deionized water (Hydrolab, Straszyn, Poland). Test substance standards amitriptyline hydrochloride solution (1 mg/mL), mianserin hydrochloride solution (1 mg/mL), duloxetine hydrochloride solution (1 mg/mL), mirtazapine solution (1 mg/mL), N-desmethylmirtazapine solution (1 mg/mL), sertraline hydrochloride solution (1 mg/mL), norsertraline hydrochloride solution (100 μg/mL), citalopram hydrobromide solution (1 mg/mL), N-desmethylcitalopram hydrochloride solution (1 mg/mL), and O-desmethylvenlafaxine hydrochloride were purchased from Sigma-Aldrich (St. Louis, MO, USA). Venlafaxine hydrochloride was supplied by Moehs Iberica SL (Barcelona, Spain). Chlordiazepoxide, the internal standard (IS), was purchased from Polfa Tarchomin (Warsaw, Poland).

### 2.2. Standard Solutions

Standard solutions of venlafaxine hydrochloride and O-desmethylvenlafaxine hydrochloride at a concentration of 1 mg/mL were prepared by dissolving 10 mg of the substance in 10 mL of methanol. A working solution (amitriptyline hydrochloride, mianserin hydrochloride, duloxetine hydrochloride, mirtazapine, N-desmethylmirtazapine, sertraline hydrochloride, norsertraline hydrochloride, citalopram hydrobromide, N-desmethylcitalopram hydrochloride, O-desmethylvenlafaxine hydrochloride, and venlafaxine hydrochloride), at a concentration of 10 μg/mL, was prepared by mixing 40 μL each of analyte stock solutions (1 mg/mL) and diluting it with methanol to a volume of 4 mL. Another working solution of 1 μg/mL was prepared by mixing 40 μL each of the analyte solutions (100 μg/mL) and diluting it with methanol to a volume of 4 mL. A chlordiazepoxide (IS) stock solution of 1 mg/mL was prepared by dissolving 10 mg of the substance in 10 mL of methanol. A working solution of 1 μg/mL was prepared by diluting the stock solution in methanol. The internal standard and the 1 μg/mL and 10 μg/mL working solutions were stored at −21 °C.

### 2.3. UHPLC-DAD Analysis

A Nexera XR UHPLC liquid chromatograph (Shimadzu, Kyoto, Japan) equipped with an LC-30AD pump, CTO-20AC thermostat, CBM-20Alite control system, SIL-30AC autosampler, SPD-M30A UV-VIS detector with diode array, and SPD-M30A high-sensitivity-measuring cell (85 mm) was used for chromatographic separation. Chromatographic separation was carried out on a Luna Omega 3 µm column (LC Column 100 × 3.0 mm ID) with Polar C18 100 fill and precolumn (Polar C18, 4 × 2.0 mm ID). In contrast, the mobile phase was a binary system consisting of water with formic acid (pH 3.5) (solvent A) and acetonitrile (solvent B), with a gradient program starting from 90:10 (*v*/*v*, solvent A/solvent B) to 35:65 (*v*/*v*, solvent A/solvent B).

### 2.4. Collection and Sample Preparation

Oral fluid samples were collected using Salivettes. The healthy volunteers were required to not consume beverages or meals for at least half an hour before sampling, and to rinse their mouths with water 10 min before sampling. The swabs were placed in the mouth for about 2 min and centrifuged at 8000 rpm for 5 min, with the resulting oral fluid frozen and stored at −21 °C until analysis.

### 2.5. Supported Liquid Extraction

Thawed saliva samples were centrifuged at 8000 rpm for 5 min, and 0.5 mL of fluid was collected and transferred to polypropylene tubes. The samples were then diluted 1:1 with water, 100 µL solution (1 µg/mL) of internal standard (the final concentration was 200 ng/mL), and an appropriate number of analyte working solutions: 50, 100 μL (100 ng/mL); 25, 50, 150 μL (1 μg/mL); 25 and 50 μL (10 μg/mL) were added. The composition and preparation of the analyte working solutions are described in Section 2.2. The prepared samples were shaken for 20 min and centrifuged at 8000 rpm. The resulting supernatant was applied to Isolute SLE+ columns. A vacuum pulse initiated the loading of the sample onto the column. Then, for full absorption and formation of the extraction layer, the samples were placed on the sorbent for 5 min. After this time, a dichloromethane elution solution with isopropanol (95:5) was applied twice to the columns. The eluate was evaporated to dryness, and the residue was dissolved in 100 μL of acetonitrile: water mobile phase with formic acid (0.1%) (10:90 *v*/*v*).

### 2.6. Method Validation

The validation process was performed in accordance with the EMA guidelines [28].

#### 2.6.1. Linearity

The linearity of the method was tested in the concentration range of 10–1000 ng/mL by preparing four calibration curves on four consecutive days. Working solutions of analytes were added to 0.5 mL of saliva to produce final concentrations of 10, 20, 50, 100, 300, 500, and 1000 ng/mL and the internal standard at 200 ng/mL. A calibration curve was determined from the relationship of the ratio of analyte peak area to IS area as a function of analyte concentration.

#### 2.6.2. Intra- and Inter-Day Precision

Intra- and inter-day precision were determined for three analyte concentrations (low QC, medium QC, and high QC). For intra-day precision, each concentration was analyzed five times within one day. In contrast, inter-day precision was determined by performing five analyses of each concentration for four consecutive days (n = 20). The precision of the method was expressed by the coefficient of variation (% CV), with an acceptable value taken as ≤15%.

#### 2.6.3. Limits of Detection and Quantification

The limit of detection (LOD), which is the lowest concentration or amount of analyte whose signal is three times higher than the background signal, was determined by analyzing loaded saliva samples with increasing analyte content. Each concentration was analyzed three times.

The limit of quantification (LOQ), which represents the lowest concentration or amount of analyte whose signal is ten times higher than the background signal and can be determined with a precision of CV < 20% and analyte recovery of ±20%, was determined based on five replicates. The definitions of LOD and LOQ correspond to the EMA guidelines [28].

#### 2.6.4. Selectivity

To determine the selectivity of the method, chromatograms of extracts of 10 blank saliva samples from healthy volunteers were analyzed. In the absence of interferences caused by the presence of endogenous compounds, the method was defined as selective.

#### 2.6.5. Absolute Recovery and Extraction Recovery

The recovery and extraction efficiency of the method was determined for two analyte concentrations (medium QC and high QC) by analyzing each analyte six times. By comparing the peak areas of the extracted analytes with the peak areas of the six neat standards, absolute recovery was determined. An acceptable result was considered to be a value exceeding 50% of the average value of the six neat standards at each concentration.

By comparing the areas of the extracted analytes to those obtained from ten blank post-spiked samples, extraction recovery was determined. An acceptable result was considered to be a value exceeding 50% of the average value of the post-spiked samples at each concentration. In both cases, extraction efficiency and the adequate recovery of analytes exceeded 50%, meeting EMA guidelines [28].

#### 2.6.6. Stability

The stability of the analytes in the biological matrix was tested by storing samples at +8 °C and performing a freeze–thaw test at −21 °C. In both cases, three concentrations of analytes were tested (low QC, medium QC, and high QC). For each concentration, 2 mL of saliva was placed in 5 tubes and loaded with the appropriate amount of standard analyte mixture solution. After mixing, 0.5 mL of liquid was taken, extracted, and subjected to chromatographic analysis. The remainder of the sample was placed in the refrigerator or frozen, and analyzed on subsequent days.

The stability of analyte extracts stored in the autosampler at 15 °C for 72 h was also determined by repeated chromatographic analysis of the samples. Those compounds whose concentrations decreased less than 15% regardless of their storage conditions were considered stable.

### 2.7. Clinical Application

In order to determine the applicability of the developed method in clinical studies, saliva samples were collected from women (n = 14) treated with the tested compounds in single and multiple drug treatments. Saliva samples were collected from female patients of the Hospital for the Nervous and Mentally Ill in Starogard Gdanski (Poland), without stimulation, by directly placing the oral fluid in a tube. The average age of the patients was 52 years. Samples were collected in the morning, around 10 am, and then frozen. Due to the multiple-drug treatment, the time elapsed since the administration of each drug was not taken into account during sampling. Prior to analysis, samples were thawed, and 0.5 mL of saliva was collected, extracted, and analyzed chromatographically. The study protocol was approved by the ethical committee of the Medical University of Gdansk, Poland (NKBBN/393/2021).

## 3. Results

While antidepressants are used primarily in the treatment of depression, they are also used in the treatment of mood, sleep, eating, and anxiety disorders, as well as for sexual disorders and pain complaints. The multitude of indications for their use means that they can also easily be abused and can lead to psychological dependence on the drugs taken. In addition, like any drug, they can cause more or less severe side effects, which can result in the patient abandoning treatment without prior consultation with the doctor. Hence, it is important to control use during therapy and after the patient no longer needs it for any symptoms of psychological dependence.

### 3.1. Chromatographic Separation

**Figure 2:**
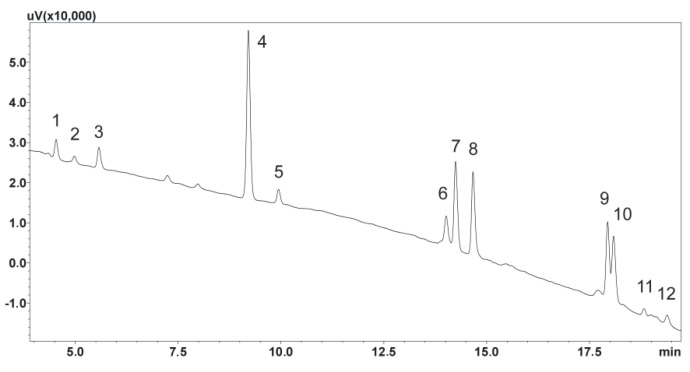
Chromatogram of the standard solutions obtained by optimized UHPLC. (1) N–desmethylmirtazapine; (2) O–desmethylvenlafaxine; (3) mirtazapine; (4) chlordiazepoxide, IS; (5) venlafaxine; (6) mianserin; (7) N–desmethylcitalopram; (8) citalopram; (9) duloxetine; (10) amitriptyline; (11) N–desmethylsertraline; (12) sertraline.

Before analyzing the saliva samples, chromatographic separation was optimized. Some of the compounds, such as duloxetine and amitriptyline, have very similar retention times due to their similar physicochemical properties. Similarly, mianserin, citalopram, and its metabolite (N-desmethylcitalopram) require special attention when selecting appropriate chromatographic separation parameters. To optimize the chromatographic separation process, the analysis time, mobile phase composition, and flow rate were adjusted. In the first step, a fifteen-minute analysis was used, with a mobile phase flow rate of 0.5 mL/min, a linear increase in solvent B from 20 to 65%, and the addition of 0.04% formic acid. Under these conditions, not all analytes separated. Therefore, the individual separations of compounds were adjusted to a flow rate of 0.7 mL/min, the addition of formic acid to 0.1%, and the analysis time to 20 min. Under these conditions, not all analytes fully separated either. Finally, it was decided to set the mobile phase flow rate at 0.8 mL/min and to extend the linear concentration of solvent B by 23 min from 10% to 40% (Figure 2), and the concentration of formic acid in solvent A to 0.1%. 

The next step was to choose the appropriate wavelength to observe the peaks during chromatographic separation. The most optimal wavelength at which the peak areas of the analytes were analyzed turned out to be 232 nm. Chlordiazepoxide was chosen as the internal standard due to its physicochemical properties, as well as its good absorption of radiation at the mentioned wavelength. This is a benzodiazepine derivative, which is no longer used in medical treatment, which provided an additional advantage, as there is no possibility of the accidental presence of this substance in the patient’s saliva.

### 3.2. Extraction Procedure

**Figure 3:**
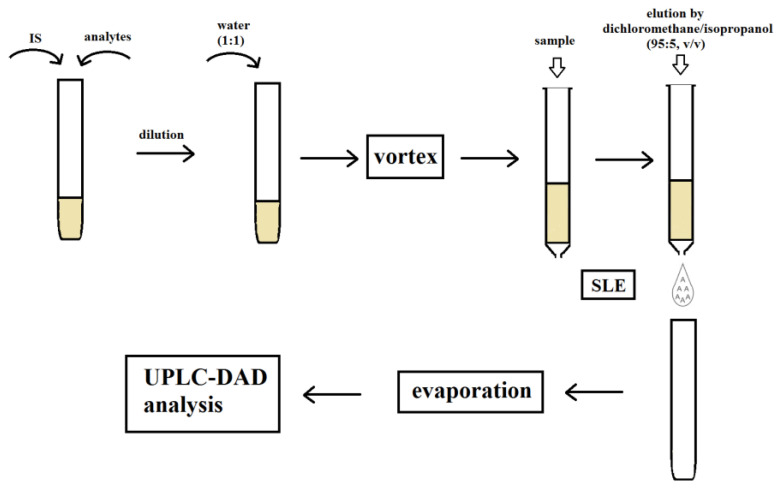
Final extraction procedure for antidepressant isolation.

**Figure 4:**
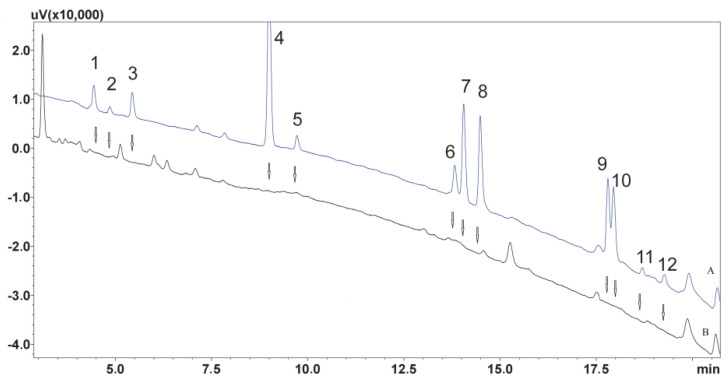
(A) Chromatogram of saliva sample extract obtained spiked with 100 ng/mL; (B) chromatogram of blank saliva sample extract. (1) N–desmethylmirtazapine; (2) O–desmethylvenlafaxine; (3) mirtazapine; (4) chlordiazepoxide, IS; (5) venlafaxine; (6) mianserin; (7) N–desmethylcitalopram; (8) citalopram; (9) duloxetine; (10) amitriptyline; (11) N–desmethylsertraline; (12) sertraline.

The optimization of the extraction process is a key element when isolating analytes. Therefore, in the next stage of the study, the effect of the solution used to dilute the sample on the binding strength of the analytes to the sorbent was determined. For this purpose, samples were diluted with 0.1% formic acid solution, water, or 0.5 M ammonia solution. The effect of the solution leaching the analytes from the columns was also investigated. Three mixtures of solutions that do not mix with water and have high lipophilicity were chosen for the study: dichloromethane, dichloromethane mixed with isopropanol (95:5, *v*/*v*), and ethyl acetate. Thus, nine procedures were created and used to isolate the compounds (Table 1). The best results were obtained using water to dilute the samples and dichloromethane in a mixture with isopropanol to elute the analytes from the columns. The developed procedure both allowed satisfactory purification of the extracts and ensured high recoveries, especially for parent compounds characterized by higher lipophilicity than their metabolites. Next, the effect of solvent volume on the efficiency of the elution of analytes from the column was studied. The manufacturer recommends using 5 mL of eluting solvent twice. Finally, the volume of 4.5 mL used twice allowed it to maintain high extraction efficiency and adequate recovery of analytes meeting EMA guidelines, i.e., exceeding 50%. A diagram describing the final extraction procedure is shown in Figure 3. Example chromatograms showing extracts of both unloaded and loaded saliva samples obtained using the developed extraction procedure are shown in Figure 4.

### 3.3. Method Validation

The SLE method requires the use of an aqueous sample and a solvent significantly different in polarity from water when leaching analytes from the column. To avoid over-diluting the sample with the methanol used in preparing the stock solutions of the analytes, the working solution was prepared as a mixture with a concentration of 10 μg/mL of all the analyzed compounds. In contrast, the 100 ug/mL working solution was prepared as a mixture of analytes except for norsertraline, whose 100 μg/mL stock solution was added directly to the loaded samples. For most compounds, the LOQ was determined at 10 ng/mL. The exceptions were citalopram, whose LOQ was 4 ng/mL, and mianserin, mirtazapine, and venlafaxine, for which the LOQ was set at 8 ng/mL. 

Calibration curves for all compounds tested were determined in the range of 10–1000 ng/mL. In all cases, the method was linear, with R^2^ > 0.99. Data on calibration curves and method validation are summarized in Table 1. The results obtained indicate that the developed method is selective and precise. This is confirmed by the CV value, which did not exceed 15% for all tested compounds, either in intra- or inter-day precision.

### 3.4. Extraction and Absolute Recovery

Extraction efficiency was determined by comparing the peak areas of the extracted analytes with the peak areas of blank post-spiked samples. For the two concentrations of analytes tested (150 and 740 ng/mL), the extraction method was suitable, and its efficiency exceeded 50%. The lowest efficiency was obtained by isolating duloxetine, which did not exceed 60% in any of the cases tested. It was also observed that higher recovery values were obtained for the parent compounds. This may be due to the higher lipophilicity of the parent compounds than their metabolites. Detailed data on extraction yields and absolute recoveries are presented in Table 2.

### 3.5. Stability

The stability of the analytes was tested both in the biological material by storing it in the refrigerator and freezing it in the freezer, and while holding the extracts in the autosampler. The results of the tests carried out are summarized in Table 3. The values shown are the analyte concentrations expressed as a percentage of the analyte content determined on the first day of a given analysis. Based on the results obtained, it can be concluded that all tested compounds were stable regardless of their storage conditions. All analytes met EMA guidelines and did not degrade beyond 15% of the initial concentration value. The exception was N-desmethylcitalopram, whose concentration during storage in the autosampler fell slightly below 80% for a concentration of 15 ng/mL. For the remaining concentrations (150 and 740 ng/mL), the percentage of analyte compared to the initial content decreased by less than 20%, and was 82.62% and 84.05%, respectively.

### 3.6. Clinical Application

**Figure 5:**
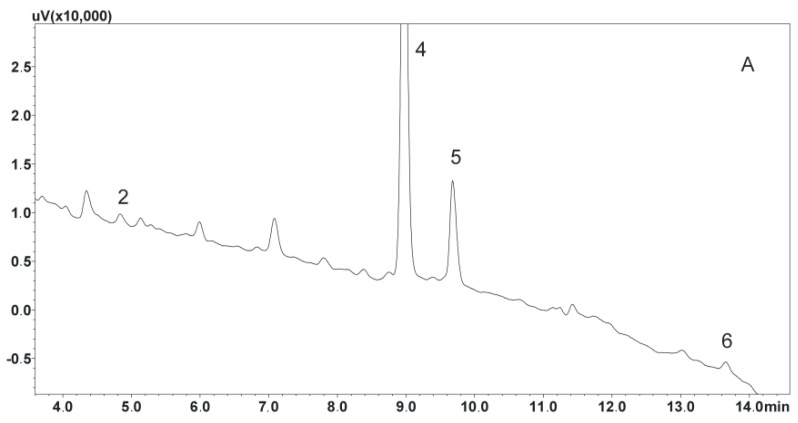

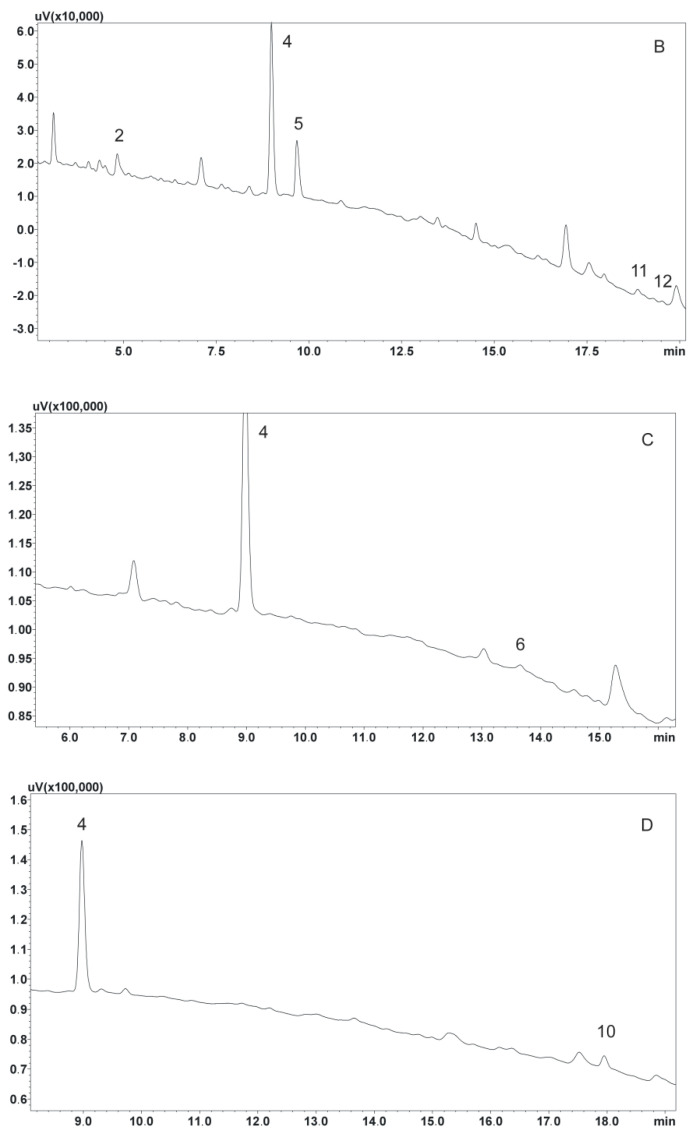
Chromatogram of saliva extracts of patients treated with studied compounds: (**A**) venlafaxine and mianserin, patient 8 (47 years); (**B**) venlafaxine and sertraline, patient 10 (57 years); (**C**) mianserin, patient 4 (28 years); (**D**) amitriptyline, patient 13 (55 years). (1) N–desmethylmirtazapine; (2) O–desmethylvenlafaxine; (3) mirtazapine; (4) chlordiazepoxide, IS; (5) venlafaxine; (6) mianserin; (7) N–desmethylcitalopram; (8) citalopram; (9) duloxetine; (10) amitriptyline; (11) N–desmethylsertraline; (12) sertraline.

The developed method was used to determine the salivary concentration of antidepressants in the saliva of 14 female patients using the studied preparations both in mono- and polytherapy. The mean age of the patients was 52 years. Details of the therapy used and the doses of the drug are shown in Table 4. In addition, the concentrations of the drug determined and its metabolite in the saliva sample are also included. The obtained results confirmed that the developed method makes it possible to detect and determine the tested compounds in all analyzed samples. Example chromatograms of the patients’ saliva extracts are shown in Figure 5.

## 4. Discussion

The novel use of SLE for the isolation of antidepressants and their metabolites from saliva allowed the simultaneous determination of seven antidepressants and four of their metabolites. The developed method was characterized by linearity in the concentration range of 10–1000 ng/mL (R^2^ > 0.99). In addition, the precision of the determinations for all tested concentrations did not exceed 15%. The determined LOQ was low and did not exceed 10 ng/mL for the parent compounds (amitriptyline, duloxetine, and sertraline) and all tested metabolites. For citalopram, the LOQ was the lowest, at 4 ng/mL. For the other compounds (mianserin, mirtazapine, and venlafaxine), the LOQ was 8 ng/mL. The literature data indicate that when LC-MS/MS is used, the LOQ can be as low as 2 ng/mL for venlafaxine, citalopram, amitriptyline, and sertraline [10]. This is related to the type of detection used.

There is a lack of information in the literature on the therapeutic concentration ranges of the tested drugs in saliva. Therefore, the range in linearity of the method was chosen based on the few existing reports on the determination of concentrations in patient saliva and therapeutic concentrations in blood. It is usually assumed that the drug concentration in saliva corresponds to 10% of the blood concentration [29]. In addition, the pKa of the compound and the degree of binding to blood proteins were included, as most studies on psychoactive drugs indicate that the correlation between saliva and blood concentrations is high for a non-protein-bound compound [29]. The literature data indicate that the therapeutic reference range for amitriptyline should be between 80 and 200 ng/mL; for citalopram 50–110 ng/mL; for venlafaxine and its metabolite it should be between 100 and 400 ng/mL; for duloxetine 30–120 ng/mL; for mianserin 15–70 ng/mL; for mirtazapine 30–80 ng/mL; and for sertraline 10–150 ng/mL [1].

To date, the tested antidepressant compounds have been determined separately in saliva, and SPE has been used most often for their isolation [10,11,15,16,17,18,19]. SLE is a modification of LLE, which usually has inferior process yields. However, in this study, SLE extraction yields were comparable to those using SPE. For amitriptyline extraction, recovery ranged between 110.04% and 76.08% for 150 and 740 ng/mL, respectively. In contrast, absolute recovery was 98.27% and 78.97% for the same concentrations.

The literature data indicate that amitriptyline was isolated from saliva with similar efficiencies to using SPE, of around 70% [15,16] or above 90% [11,18]. Using SLE, the yields for citalopram, mirtazapine, and venlafaxine were at similar levels to those obtained using SPE [18].

Another antidepressant drug isolated from saliva using SLE is sertraline, with an extraction efficiency not above 80%, and between 57.93% and 75.38% for its metabolite. The literature data on the isolation of this compound from saliva by SPE with the use of mix-mode columns indicated that extraction yields to the solid phase can also vary depending on the study; 46.5–74.5% [11], 50% [15], and 83.2% [18], which is comparable to the extraction efficiency of SLE. However, there are no data on process yields for the metabolite sertraline.

There is no information in the literature on the isolation of mianserin from saliva. In this study, the extraction efficiency of mianserin was about 100%. SLE as a method of isolation of antidepressant compounds produces better results for parent compounds than metabolites. The exception was duloxetine, for which the extraction efficiency was lower and did not exceed 60%. Only one study on the isolation of this compound from saliva can be found in the literature, and it was conducted using SPE [18].

In order to examine the feasibility of using SLE in diagnosis, this study tested saliva samples of patients undergoing antidepressant treatment. The developed method made it possible to determine the levels of all the tested compounds and their metabolites. This study showed that the content of venlafaxine in saliva used at a dose of 75 mg and 150 mg was in the range of 32.04–76.30 ng/mL and 201.86–486.46 ng/mL, respectively. In contrast, the concentration of its metabolite in saliva ranged between 43.45 and 289.28 ng/mL. The results obtained are consistent with the literature data for both venlafaxine and its metabolite [7,8,10,12,13,18]. The concentrations of citalopram and its metabolite determined in the saliva of two patients were 57.69 and 55.29 ng/mL and 162.46 and 51.78 ng/mL for the parent compound and its metabolite, respectively. Again, the literature data show similar results [7,9,10,12,17,18]. In the case of sertraline, the range of concentrations of the parent drug and metabolite in saliva was 35.42–81.37 ng/mL and 12.25–148.38 ng/mL, respectively, which is consistent with the literature data [9,18]. Examined saliva samples of patients using amitriptyline at a dose of 100 mg per day showed that the analyte content was 92.29 and 73.25 ng/mL. The available literature data indicate the content of amitriptyline at a level of several ng/mL [18,21] or a concentration slightly exceeding 30 ng/mL when the drug was used at a dose of 50 mg per day [10]. This study showed that the level of mianserin used at a dose of 60 or 40 mg per day ranged from 12.48 to 62.83 ng/mL in the saliva samples tested. However, there is no information in the literature on the concentration of mianserin in human saliva.

## 5. Conclusions

Drug concentration monitoring is commonly performed in blood. However, due to the numerous inconveniences associated with taking blood samples, it is important to look for alternative diagnostic materials. One such material is saliva. While there is a lack of documented information on therapeutic reference ranges of drugs in saliva, and procedures for the isolation of analytes are not standardized, it is desirable to develop effective methods using saliva as a diagnostic material and formalize its use in TDM. To date, there is no information in the available literature on the use of SLE for the isolation of antidepressants. Given their lipophilic properties and the high water content of saliva, SLE may be an alternative to the most commonly used SPE, as evidenced by the results of this study.

The developed method of SLE for the isolation of analytes made it possible to determine antidepressants (amitriptyline, mianserin, duloxetine, mirtazapine, sertraline, citalopram, and venlafaxine) and their metabolites (N-desmethylmirtazapine, norsertraline, N-desmethylcitalopram, and O-desmethylvenlafaxine) in saliva. The SLE extraction method allowed the isolation of all tested compounds from a small volume of biological material (0.5 mL). According to the available literature, SLE has not yet been used to isolate antidepressant compounds and their metabolites from saliva. The obtained extracts were characterized by high purity, and chromatograms lacked matrix-derived interferents. The developed extraction method was characterized by high efficiency, although it showed better efficiency for the parent compounds than for their metabolites. 

Based on the results, it can be concluded that the extraction method had good linearity for all tested compounds (R^2^ > 0.99). Low limits of quantification were achieved, at under 10 ng/mL for any of the tested compounds. In addition, the CV for intra- as well as inter-day precision was also low and did not exceed 15% for any of the compounds tested. 

The developed extraction method made it possible to determine antidepressant drugs along with their metabolites in all tested saliva samples from patients treated with these drugs. Thus, it can be concluded that this method can be used to monitor the levels of antidepressants and control their intake by patients in a readily available biological material such as saliva.

## Figures and Tables

**Table 1:** Procedures used to examine the efficacy of supported liquid extraction (SLE).

Procedure	Dilution Solvent (0.5 mL)	Elution Solvent (2 × 4.5 mL)	Conclusions
1.	0.1% formic acid solution	dichloromethane	numerous contaminants from the biological matrix, invisible substance peaks at 20 ng
2.		dichloromethane/isopropanol (95:5, *v*/*v*)	satisfactory results, but poor elution of sertraline and its metabolite
3.		ethyl acetate	numerous contaminants from the biological matrix
4.	Water	dichloromethane	impurity peak at IS retention time, poor elution of analytes
5.		dichloromethane/isopropanol (95:5, *v*/*v*)	satisfactory results, without impurity peaks at analyte retention times, satisfactory elution of all analytes, better than in procedure 8
6.		ethyl acetate	numerous contaminants from the biological matrix
7.	0.5 M ammonia solution	dichloromethane	impurity peaks at analyte retention times, poor elution of analytes
8.		dichloromethane/isopropanol (95:5, *v*/*v*)	satisfactory results, without impurity peaks at analyte retention times, satisfactory elution of all analytes
9.		ethyl acetate	numerous contaminants from the biological matrix

**Table 2:** Calibration curves (linearity in the range 10–1000 ng/mL) and validation parameters for the developed method.

Analyte	R^2^	Slope	Intercept	LOQ (ng/mL)	Intra-Day CV (%)			Inter-Day CV (%)			Extraction Recovery (%)		Absolute Recovery (%)	
					15	150	740	15	150	740	150	740	150	740
Amitriptyline	0.9988	0.0025	−0.0385	10	8.38	6.88	3.33	14.53	7.58	5.30	110.04	76.08	98.27	78.97
Citalopram	0.9992	0.0021	0.0066	4	13.40	4.56	3.58	13.42	5.57	4.19	107.73	81.94	104.35	90.86
N-desmethylcitalopram	0.9987	0.0015	−0.0049	10	10.38	8.69	4.16	14.76	8.81	4.11	61.02	58.83	59.25	60.45
Duloxetine	0.9977	0.0016	−0.0136	10	9.52	7.51	3.22	11.64	10.68	3.84	55.39	59.51	57.94	59.35
Mianserin	0.9992	0.0009	−0.0053	8	8.80	5.68	2.93	14.75	11.57	4.00	107.09	93.36	108.54	93.15
Mirtazapine	0.9980	0.00075	−0.0069	8	11.00	5.81	3.29	12.95	7.36	4.66	101.48	93.69	103.54	94.06
N-desmethyl-mirtazapine	0.9977	0.0005	−0.0117	10	2.50	6.66	3.52	12.35	10.20	3.40	62.34	63.13	53.72	62.95
Sertraline	0.9979	0.0003	−0.0028	10	10.40	6.63	3.10	14.71	9.72	4.28	74.61	75.25	79.16	78.21
N-desmethylsertraline	0.9979	0.0002	−0.0001	10	10.31	7.00	4.03	11.64	7.71	4.72	75.38	57.93	66.47	59.77
Venlafaxine	0.9986	0.0005	0.0008	8	11.40	8.43	4.33	14.89	8.86	4.78	108.17	98.79	101.23	97.49
O-desmethylvenlafaxine	0.9989	0.0002	−0.0031	10	11.26	8.17	4.02	14.97	9.45	4.47	67.48	70.91	85.34	67.90

**Table 3:** Stability study of analytes in spiked saliva.

Conditions	−21 °C (Freeze-Thaw Cycles)			8 °C (Fridge)			15 °C (Autosampler)		
Concentration (ng/mL)	15	150	740	15	150	740	15	150	740
Analyte	The percentage of analyte concentrations in relation to the amount determined on the first day of the analysis.								
Amitriptyline	95.19	97.65	100.87	97.97	101.28	95.30	95.19	100.89	99.80
Citalopram	96.36	101.57	101.36	102.53	96.84	97.47	98.35	96.45	98.58
N-desmethylcitalopram	102.14	101.90	100.79	97.64	102.71	95.67	79.36	82.62	84.05
Duloxetine	101.94	96.08	100.52	98.12	98.80	100.66	96.50	91.66	85.35
Mianserin	102.31	101.17	101.13	101.37	98.58	100.47	101.24	97.82	104.05
Mirtazapine	99.53	96.29	101.25	91.50	101.62	101.31	85.48	99.05	100.81
N-desmethylmirtazapine	96.35	102.22	100.50	97.16	100.31	98.01	98.83	95.31	91.12
Sertraline	96.73	96.54	100.65	104.54	100.52	98.46	98.22	101.68	97.95
N-desmethylsertraline	100.05	98.37	102.19	97.66	94.54	98.49	96.44	103.86	98.61
Venlafaxine	98.56	98.30	93.29	101.41	101.91	100.48	98.74	97.56	92.98
O-desmethylvenlafaxine	101.49	96.61	102.52	101.05	98.52	97.00	89.55	94.11	90.04

**Table 4:** The concentrations of antidepressants and their metabolites in saliva samples (drug doses in mg/day used by the patients are given in the parentheses).

Saliva Sample	Patient Age	Concentration (ng/mL)/Dose (mg/Day)							
		Amitriptyline	Citalopram	N-Desmethyl- Citalopram	Mianserin	Sertraline	N-Desmethyl- Sertraline	Venlafaxine	O-Desmethyl- Venlafaxine
1	53				42.16 (40)				
2	59					35.42 (50)	44.44		
3	56					81.37 (100)	77.23		
4	28				51.98 (60)				
5	47							76.30 (75)	122.82
6	49							48.01 (75)	117.50
7	65	92.29 (100)							
8	47				62.83 (60)			294.42 (150)	43.45
9	59					50.92 (50)	12.25	201.86 (150)	121.72
10	57					42.96 (50)	148.38	486.46 (150)	289.28
11	46		57.69 (20)	55.29	57.83 (60)				
12	48		162.46 (20)	51.78	48.14 (60)				
13	55	73.25 (100)							
14	56				12.48 (40)			32.04 (75)	50.73

## Data Availability

The data presented in this study are available on request from the corresponding author.
